# Longitudinal Changes in Factors Associated with Walking Independence at Hospital Discharge in Patients with Stroke: A Retrospective Study

**DOI:** 10.3390/jcm13237184

**Published:** 2024-11-27

**Authors:** Ryosuke Yamamoto, Shin Murata, Shun Sawai, Shoya Fujikawa, Yusuke Shizuka, Takayuki Maru, Kotaro Nakagawa, Hideki Nakano

**Affiliations:** 1 Graduate School of Health Sciences, Kyoto Tachibana University, 34 Yamada-cho, Oyake, Yamashina-ku, Kyoto-shi 607-8175, Kyoto, Japan; h901123013@st.tachibana-u.ac.jp (R.Y.); murata-s@tachibana-u.ac.jp (S.M.); sawai.neuroreha@gmail.com (S.S.); fujikawa.pt@gmail.com (S.F.); shizuka.reha@gmail.com (Y.S.); 2 Department of Rehabilitation, Tesseikai Neurosurgical Hospital, 28-1 Nakanohon-Machi, Shijonawate-shi 575-8511, Osaka, Japan; 3 Department of Physical Therapy, Faculty of Health Sciences, Kyoto Tachibana University, 34 Yamaca-cho, Oyake, Yamasina-ku, Kyoto-shi 607-8175, Kyoto, Japan; maru@tachibana-u.ac.jp (T.M.); nakagawa-k@tachibana-u.ac.jp (K.N.); 4 Department of Rehabilitation, Kyoto Kuno Hospital, 22-500 Honmachi, Higashiyama-ku, Kyoto-shi 605-0981, Kyoto, Japan; 5 Department of Rehabilitation, Junshinkai Kobe Hospital, 868-37 Kozukadai, Tarumi-ku, Kobe-chi 655-0008, Hyogo, Japan; 6 Nagashima Neurosurgery Rehabilitation Clinic, 1st and 2nd floor Niitaka Clinic Center Building, 2-3-2 Niitaka, Yodogawa-ku, Osaka-shi 532-0033, Osaka, Japan

**Keywords:** assessment of walking independence, stroke, longitudinal change, Mini-BESTest, rehabilitation

## Abstract

**Background/Objectives**: Patients with stroke usually have long-term residual gait disability. However, temporal changes in factors associated with gait independence in these patients at the time of hospital discharge have not been clarified. This study aimed to determine changes over time in factors associated with gait independence in patients with stroke at the time of hospital discharge. This would predict that factors associated with the level of walking independence in patients with stroke at discharge from the hospital would show different results depending on the changes over time post-stroke onset. **Methods**: This retrospective observational study used data from the medical records of patients with stroke with unilateral supratentorial lesions who were admitted and rehabilitated at Tesseikai Neurosurgical Hospital between October 2020 and July 2024. The Functional Ambulation Category (FAC), Stroke Impairment Assessment Set-lower extremity motor items, Trunk Control Test, Mini-Balance Evaluation Systems Test (Mini-BESTest), and Functional Independence Measure cognitive items were assessed monthly for 3 months post-stroke onset. Participants were classified into independent and non-independent walking groups using the FAC. Logistic regression analysis was performed with walking independence at discharge and other assessment indicators as the dependent and independent variables, respectively, to identify factors influencing walking independence at discharge. Independent variables were entered by month from 1 to 3 months. **Results**: Logistic regression analysis revealed that Mini-BESTest scores at 2 and 3 months post-stroke onset were significantly associated with walking independence at discharge (*p* < 0.05). **Conclusions**: This study suggests the importance of assessing the Mini-BESTest scores over time, starting at 2 months post-stroke onset, when determining walking independence in patients with stroke. Providing balance training to patients with low Mini-BESTest scores between 1 and 3 months post-stroke onset may contribute to improved walking independence at discharge.

## 1. Introduction

Stroke affects approximately 15 million individuals annually worldwide and is the second and third leading cause of death and disability, respectively [1]. Research indicates that 40% of patients with stroke exhibit moderate functional disability, while 15–30% experience severe impairment [2].

Approximately 30% of patients with stroke require assistance for ambulation 6 months post-stroke onset [3] and need support in activities of daily living [4]. Consequently, stroke results in long-term physical impairment, necessitating interventions to increase the functional capacity of affected individuals. Gait impairment in patients with stroke significantly impacts their independence in activities of daily living, underscoring the importance of gait rehabilitation in stroke recovery. Additionally, gait disturbances in patients with stroke can be attributed to multiple complex factors, including motor and sensory impairments, spasticity, and equilibrium deficits [5].

The incidence of falls within 6 months of stroke onset ranges from 25% to 37% [6], resulting from various functional factors, including muscle weakness, impaired balance, compromised walking ability, and cognitive deficits [6]. Therefore, multiple functional domains need to be addressed in patients with stroke to increase their ambulatory ability and enable them to walk without falling during daily activities. In addition to the health aspects related to the patients, gait disturbance is associated with increased caregiver burden, higher healthcare costs, and greater utilization of social resources [7,8], making gait disturbance in patients with stroke of considerable societal concern. Improving multiple functions and walking ability during stroke rehabilitation is vital. However, not all patients with stroke achieve independent ambulation, with only approximately 60% achieving independent ambulation [9].

Accurately predicting the level of walking independence at discharge and implementing interventions tailored to each patient’s ability and expected functional improvement is important in stroke rehabilitation. Factors influencing gait independence have been investigated to predict gait independence in patients with stroke. Physical function upon hospital admission reportedly correlates with the level of walking independence at discharge [1,10,11]. The Stroke Impairment Assessment Set-lower extremity (SIAS-LE) motor items immediately post-stroke onset have been identified as predictors of walking independence at discharge [1]. Similarly, the Trunk Impairment Scale (TIS) score at the same time point has been shown to predict ambulatory independence at discharge, with a cutoff value of 12 points serving as a predictor of ambulatory independence at discharge [10,12]. The Berg Balance Scale score immediately post-stroke onset is also a predictor of ambulatory independence at discharge, with a cutoff value of 29 points predicting ambulatory independence at discharge [13]. Considering this, walking independence at discharge can be predicted based on physical function immediately post-stroke onset. In contrast, the Mini-Balance Evaluation Systems Test (Mini-BESTest) is a predictor of achieving community-level walking speed (>0.8 m/s), with a cutoff value of 18.5 points [14]. Therefore, the Mini-BESTest predicts the level of walking independence using physical function at a single time point.

Nevertheless, changes in factors associated with walking independence at hospital discharge remain unclear. A retrospective observational study has investigated changes in physical function, cognitive function, and walking ability over 6 months post-stroke onset [15]. Physical function and walking ability before and after 4 weeks of rehabilitation in patients with chronic stroke at 6 months post-stroke onset have also been previously compared [16]. However, investigating long-term changes or determining the impacts of monthly physical and cognitive functions post-stroke onset on walking independence at hospital discharge has not been possible. Therefore, this study aimed to investigate changes in hemiplegic lower limb function, trunk function, balance capacity, and cognitive function from 1 to 3 months post-stroke onset and determine how these factors relate to walking independence at hospital discharge within the same time points. Previous studies have reported continuous improvements in physical and cognitive functions and walking ability during the first 6 months post-stroke onset. Therefore, this study may also show improvement over time in the paralyzed side’s lower limb function, trunk function, balance capacity, and cognitive function between 1 and 3 months post-stroke onset. However, the functions that show significant improvement differ for each patient. Other factors may be extracted from the association of ambulatory independence at discharge with paraplegic leg function, trunk function, balance ability, and cognitive function in patients with stroke between 1 and 3 months post-stroke onset, depending on the month. Elucidating these factors may contribute to establishing a gait independence prediction model that considers the time from stroke onset. Furthermore, we propose that the potential for a specific focal point of intervention at each phase of stroke rehabilitation exists.

## 2. Materials and Methods

### 2.1. Participants

**Figure 1:**
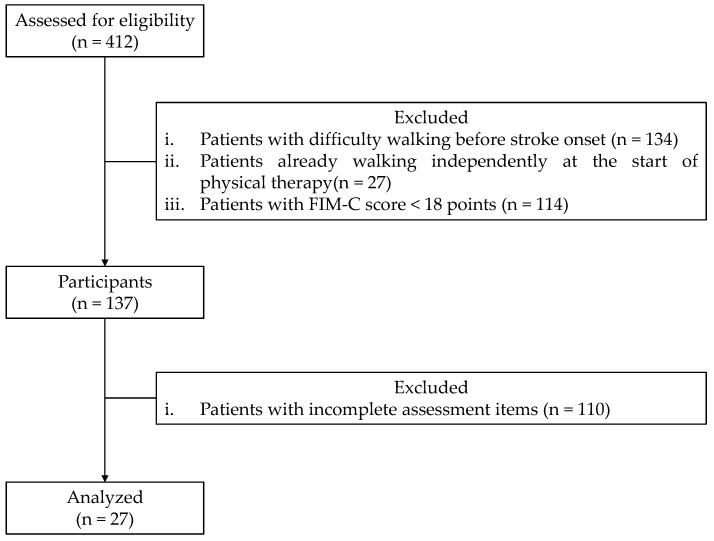
Flow diagram illustrating participant selection. FIM-C: Functional Independence Measure Cognitive Component score.

This study included 412 patients with or without osteoarthritis in the lower limbs who were hospitalized and rehabilitated for at least 3 months for unilateral supratentorial lesion stroke at the Tesseikai Neurosurgical Hospital between October 2020 and July 2024. Patients with difficulty walking pre-stroke onset, those who could walk independently at the start of physical therapy, those with a total Functional Independence Measure Cognitive Component (FIM-C) score of <18 points [17], and those with incomplete assessment items were excluded. Additionally, patients with an FIM-C score of <18 were excluded based on a previous study [17], where patients with stroke were categorized into the following three groups according to the degree of assistance they received while walking: unable-to-walk, moderate- or light-assistance, and monitored or fully independent groups. The mean FIM-C score for each group was determined, with the moderate- or light-assistance group having an FIM-C score of 18 ± 4.7 [17]. Overall, the final analysis included 27 patients (Figure 1). There were no losses to follow-up in this study. All patients who met the inclusion criteria and were not excluded based on the predefined exclusion criteria were included in the final analysis. This study was reported according to the Strengthening the Reporting of Observational Studies in Epidemiology guidelines.

### 2.2. Ethics Declarations

This study was conducted following the principles of the Declaration of Helsinki and approved by the Research Ethics Committees of Tesseikai Neurosurgical Hospital (approval number: 2023--64; approval date: 23 October 2023) and Kyoto Tachibana University (approval number: 24--30; approval date: 22 July 2024).

We employed an opt-out approach, where participants were informed about the study through public announcements and provided the opportunity to decline participation. Participants who did not opt out of the study were included. Consequently, explicit informed consent was not obtained from the participants. This study adhered to institutional guidelines available at [http://www.tesseikai.jp/about/hokatsu/, (accessed on 15 October 2024)]. Specifically, the following text was included on the website: “If you do not agree to participate in the research, or if you wish to withdraw your consent, please inform the outpatient reception or staff station, fill in the ‘Non-Consent Form for the Purpose of Research on Samples, among others,’ and submit it to the outpatient reception or staff station”. An announcement was made to ensure that patients could withdraw their consent to participate. To comply with the Personal Data Protection Law of Japan and consider personal privacy and information leakage, the data obtained in this study were anonymized according to the Guidelines for the Creation and Provision of Anonymous Data established by the Ministry of Internal Affairs and Communications.

### 2.3. Clinical Assessments

This was a retrospective observational study, and all data were collected from the medical records of the Tesseikai Neurosurgical Hospital. The data used in this study were assessed monthly from 1 to 3 months post-stroke onset [15]. Each indicator was tested and measured by the therapist in charge of each participant. All participants were assessed using the Functional Ambulation Category (FAC), SIAS-LE motor item-total score, Trunk Control Test (TCT), Mini-BESTest, and FIM-C. Assessments were conducted monthly from stroke onset to discharge.

Gait independence was evaluated using the FAC, which is an assessment index that classifies walking ability on a 6-point scale from 0 to 5 based on the degree of assistance needed. The FAC has demonstrated interrater reliability and validity in patients with stroke [18]. An FAC of 4–5 indicates independent walking [19]. The FAC score at 4 weeks post-stroke onset can predict walking ability at the community level after 6 months [18]. Therefore, in the present study, patients with FAC scores of 4–5 and 0–3 were designated as the independent and non-independent groups, respectively.

Lower limb motor function on the paraplegic side was assessed using the SIAS-LE, which comprises 22 items and has demonstrated interrater reliability and validity in patients with stroke [20]. The SIAS-LE consists of a proximal lower limb test (hip flexion test), proximal lower limb test (knee flexion test), and distal lower limb test (foot pat test) and is evaluated on a 6-point scale from 0 (no movement at all) to 5 (equal to the healthy side). Furthermore, the total SIAS-LE score ranged from 0 to 15 points, with higher values indicating better paraplegic lower limb motor function.

The trunk function was evaluated using the TCT, which comprises the following four items: turning to the paralyzed side, turning to the nonparalyzed side, rising from the supine position, and maintaining the sitting position. Notably, the TCT has been reported to be interlaterally reliable and valid in patients with stroke [21]. All subscales of the TCT are rated on a scale of 0 (unable to perform without assistance), 12 (able to accomplish but utilizing the upper limbs), and 25 (able to perform the movement normally). The total scores ranged from 0 to 100, with higher values indicating superior trunk function.

Balance was assessed using the Mini-BESTest, which is used to clarify therapeutic intervention strategies for patients with impaired balance by identifying balance ability deficits. The Mini-BESTest consists of 14 items assessing six components of balance ability (biomechanical constraints, stability limits, postural change—predictive postural control, reactive postural control, sensory function, and gait stability) and has demonstrated interrater reliability and validity in patients with stroke [22,23]. Interrater reliability and validity have also been reported in patients with stroke. Each item on the Mini-BESTest is rated on a 3-point scale from 0 (severe balance impairment) to 2 (no balance impairment), with total scores ranging from 0 to 28 and higher values indicating better balance. The lower score was used for items that evaluated the left and right sides. Previously, the relationships between the 10 m walk and balance tests were examined. The Mini-BESTest was more positively correlated with positive outcomes than the Berg Balance Scale (BBS) because it included both walking and dynamic balance tasks [14]. Specifically, the BBS was originally designed to assess balance in frail older adults and reportedly results in a ceiling effect as it does not include complex tasks to evaluate dynamic balance [14]. Therefore, the Mini-BESTest was used in this study to assess balance.

Cognitive function was assessed using the FIM-C, which comprises five items—comprehension, expression, social interaction, problem-solving, and memory—and has demonstrated interrater reliability and validity in patients with stroke [24]. The FIM-C is rated on a 7-point scale from 1 to 7, with a total score ranging from 7 to 35 and higher values indicating better cognitive function.

### 2.4. Statistical Analysis

Initially, the Shapiro–Wilk test was used to assess the normality of the data. The FAC at discharge was subsequently used to categorize participants. Participants with FAC scores of 4–5 and 0–3 were classified into independent and non-independent groups, respectively. The Mann-Whitney U test was conducted to compare participant characteristics between the independent and non-independent groups. Chi-square tests were also used to examine bias in sex ratios, stroke type, and walking independence at discharge between the two groups. A two-factor repeated-measures analysis of variance (two-way ANOVA) was used to compare changes in each assessment measure over time as follows: time since stroke onset (1, 2, or 3 months) and group (independent and non-independent groups). Two-way ANOVA was also used to confirm the differences in physical and cognitive functions between the two groups of ambulatory- and non-ambulatory independent participants. The Bonferroni post hoc correction was applied for post hoc analysis using a two-way ANOVA. Logistic regression analysis was used to examine the factors associated with walking independence at discharge for each month using decreasing variables, with the dependent variable being ambulatory or non-ambulatory independence at discharge. The independent variables were the SIAS-LE, TCT, Mini-BESTest, and FIM-C scores. Previous studies investigating changes in functional recovery over time in patients with stroke had been analyzed using two-way ANOVA [15]. Logistic regression analysis was used to identify the associated factors in a previous study investigating the relationship and impact of physical activity levels and functional recovery 12 months after stroke onset [25]. Another previous study also used logistic regression analysis to investigate which clinical assessments influence physical activity levels at 12 months post-stroke, both pre-stroke and early post-stroke [26]. Therefore, this study used two-way ANOVA to investigate changes in physical and cognitive functions over time. Logistic regression analysis was also used to examine factors affecting walking independence at discharge. Due to the high follow-up rate and the absence of missing data in this study, no sensitivity analyses were deemed necessary. All statistical analyses were performed using IBM SPSS Statistics for Windows, version 29.0 (IBM Corp., Armonk, NY, USA), with a significance level of 5%.

## 3. Results

### 3.1. Characteristics of Participants

Twenty-seven patients with stroke were included for final analysis in this study. All participants agreed to participate at each stage of the study, and no individuals declined participation. They were classified into independent (*n* = 18; male: 9, female: 9) and non-independent (*n* = 9; male: 3, female: 6) groups based on FAC at discharge. The Mann-Whitney U test revealed no significant differences in age, height, weight, length of stay, or discharge between the independent and non-independent groups (*p* > 0.05) (Table 1). Additionally, the chi-square test revealed no significant differences in sex, and stroke type at discharge between the two groups (*p* > 0.05); however, a significant difference was observed in the FAC (*p* < 0.05).

### 3.2. Results of Two-Factor Analysis of Variance

**Figure 2:**
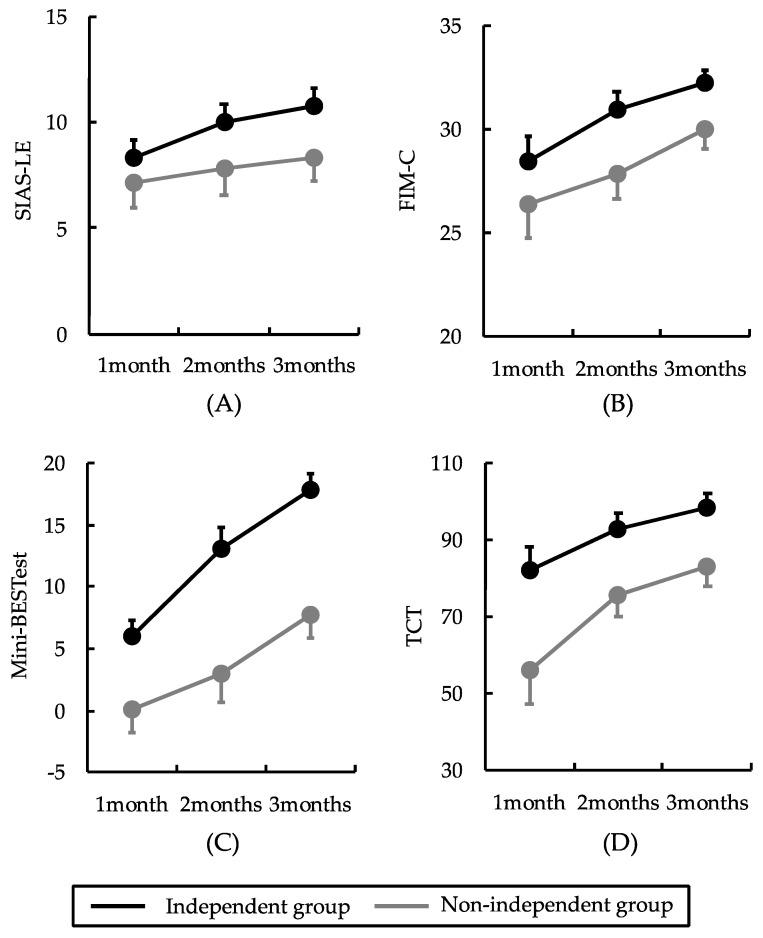
Results of the two-factor analysis of variance. No interaction effects between timing and group factors were observed. The black and gray lines indicate the independent and non-independent walking groups, respectively. Error bars are shown as standard errors. The SIAS-LE, TCT, Mini-BESTest, and FIM-C scores differed significantly over time. TCT and Mini-BESTest scores differed significantly between groups: (**A**) the SIAS-LE score significantly improved from 1 to 3 months; (**B**) TCT scores significantly improved from 1 to 3 months; (**C**) Mini-BESTest scores significantly improved from 1 to 3 months; (**D**) FIM-C scores significantly improved from 1 to 3 months. SIAS-LE: stroke impairment assessment set-lower extremity; TCT: trunk control test; Mini-BESTest: mini-balance evaluation systems test; FIM-C: functional independence measures cognitive.

A two-way ANOVA revealed no significant interactions between timing and group factors for any of the clinical assessments (*p* > 0.05). However, the SIAS-LE, TCT, Mini-BESTest, and FIM-C demonstrated significant main effects on timing (*p* < 0.05). Post-test analyses indicated that the SIAS-LE, TCT, Mini-BESTest, and FIM-C scores were significantly higher at 2 and 3 months than at 1 and 2 months, respectively (*p* < 0.05). The TCT and Mini-BESTest scores differed significantly between the groups (*p* < 0.05) but not the SIAS-LE and FIM-C scores (*p* > 0.05). Post-test analyses revealed that the TCT and Mini-BESTest scores were significantly higher in the independent group than in the non-independent group (*p* < 0.05) (Table 2 and Figure 2).

### 3.3. Results of the Logistic Regression Analysis

Logistic regression analysis indicated that clinical assessment 1 month post-stroke onset was not associated with walking independence at discharge (*p* > 0.05) (Table 3). However, at 2 and 3 months post-stroke onset, the Mini-BESTest score was significantly associated with walking independence at discharge (*p* < 0.05) (Table 4 and Table 5).

## 4. Discussion

This study aimed to determine the changes in factors associated with walking independence in patients with stroke at hospital discharge and showed that the SIAS-LE, TCT, Mini-BESTest, and FIM-C scores improved significantly from 1 to 3 months post-stroke onset. The TCT and Mini-BESTest scores were considerably higher in the independent group than in the non-independent group. Furthermore, logistic regression analysis revealed that Mini-BESTest scores at 2 and 3 months post-stroke onset were associated with walking independence at discharge. These results underscore the importance of assessing the Mini-BESTest score over time, commencing 2 months post-stroke onset, in predicting the ambulatory independence of patients with stroke at discharge.

### 4.1. Temporal Changes in Physical Function in Patients with Stroke and Comparisons of the Independent and Non-Independent Groups

Two-way ANOVA revealed no significant interaction between timing and group factors in any of the clinical assessments. The high variability of the data and small sample size may have contributed to the lack of interaction in the two-way ANOVA. In this study, the large variability in the data prevented the detection of interactions. Specifically, the measured values under each condition markedly varied, and other factors may have overshadowed the effect of the interaction; therefore, statistically significant results could not be obtained. This variability may be due to the insufficient sample size, measurement error, or the influence of external factors. In the future, we will consider a more rigorous study design and increase the sample size to reduce data variability.

We examined changes over time in the SIAS-LE, TCT, Mini-BESTest, and FIM-C scores in the independent and non-independent groups. The results demonstrated that the SIAS-LE, TCT, Mini-BESTest, and FIM-C scores improved significantly from 1 to 3 months post-stroke onset. Specifically, the TCT and Mini-BESTest scores were considerably higher in the independent group than in the non-independent group. Spontaneous neurological recovery post-stroke has been reported to occur within 3 months of onset [15]. Motor, trunk, sensory, and cognitive functions of the paralyzed side of the lower limb start to improve 1 month post-stroke onset and specifically show the greatest improvement between 1 and 3 months post-stroke onset [15]. Consequently, the SIAS-LE, TCT, Mini-BESTest, and FIM-C scores improved between 1 and 3 months post-stroke onset, likely due to neurological and functional recovery.

The SIAS-LE score improved significantly from 1 to 3 months post-stroke onset. Similar to the SIAS-LE, a previous study examining changes over time in patients with stroke using the Fugl–Meyer assessment, a measure of the paralyzed side lower-limb motor function, reported a significant improvement over time between 1 and 3 months of onset [15]. The recovery period of paraplegic motor function has also been shown to be prolonged in more severe cases, with gradual improvement observed between 3 and 6 months of onset [15]. Therefore, as in previous studies, the SIAS-LE score improved significantly at 1 and 3 months post-stroke onset.

Regarding the TCT score, it improved significantly from 1 to 3 months post-stroke onset. Similar to the TCT, a previous study examining changes over time in patients with stroke using the TIS to assess trunk function revealed significant improvement between 1 and 3 months of onset, with trunk function showing a recovery process similar to that of the paralyzed lower limb motor function. In the present study, SIAS-LE scores significantly improved from 1 to 3 months post-stroke onset, with a similar recovery process for the paralyzed side’s lower limb motor and trunk functions. The TCT score was higher in the independent group than in the non-independent group. Specifically, a study reported that patients with a TCT score ≥40 at week 1 post-stroke onset become ambulatory and independent at week 6 post-stroke onset, whereas those with a TCT score <40 did not achieve ambulatory independence until week 12 post-stroke onset [27]. Better trunk function immediately post-stroke onset has been reported to be associated with greater walking independence at discharge [28]. In our study, TCT was significantly higher in the independent group than in the non-independent group at all time points. TCT differed significantly between groups according to the two-way ANOVA post-test. Therefore, we hypothesize that trunk function in the early onset period may have influenced the difference between ambulatory independence and non-independence at discharge. Given these findings, assessing trunk function using the TCT when rehabilitating patients between 1 and 3 months post-stroke onset is important. Furthermore, training on trunk function for patients with reduced TCT scores may improve walking independence at discharge.

The Mini-BESTest score improved significantly from 1 to 3 months post-stroke onset. Similar to the Mini-BESTest, a previous study examining changes over time in patients with stroke using the BBS, a measure of balance ability, reported significant improvements between 1 and 3 months post-stroke onset [29]. In the present study, the Mini-BESTest score improved over time post-stroke onset, as observed in previous studies. Impaired balance in patients with stroke is reportedly associated with reduced motor control of the limbs, pelvis, and trunk; sensory impairment; and impaired spatial cognition [29]. A previous study has shown that paraplegic lower-limb motor, trunk, sensory, and cognitive functions significantly improve between 1 and 3 months post-stroke onset [15]. In our study, the Mini-BESTest score improved considerably between 1 and 3 months post-stroke onset, likely due to the concurrent recovery of physical, sensory, and cognitive functions. The Mini-BESTest scores were higher in the independent group than in the non-independent group. Specifically, the Mini-BESTest comprises four sections that assess predictive postural control, reactive postural control, sensory integration, and dynamic walking ability, including tasks evaluating dynamic balance and walking ability [30]. It is an assessment index with higher scores indicating better balance and walking abilities. In our study, the Mini-BESTest score significantly differed between the groups according to the two-way ANOVA post-test. This suggests that balance immediately post-stroke onset influences the difference between independent and non-independent walking at discharge. Given these findings, assessing balance using the Mini-BESTest score is important when rehabilitating patients between 1 and 3 months post-stroke onset. For patients with reduced scores on the postural control section of the Mini-BESTest, balance training may improve walking independence at discharge.

Finally, the FIM-C score improved significantly from 1 to 3 months post-stroke onset. A substantial proportion of patients with stroke present with post-stroke cognitive impairment, with a reported prevalence of 20–80% [31]. Post-stroke cognitive impairment has been observed to improve during the initial 3 months post-stroke, which is attributed to rehabilitation and neuroplasticity [32]. A previous study examining changes over time in patients with stroke using the Mini-Mental State Examination (MMSE), an assessment index of cognitive function similar to the FIM-C, reported significant improvements over time at 3, 6, and 12 months post-stroke onset [33]. Therefore, the FIM-C score improved significantly between 1 and 3 months post-stroke onset, as observed in previous studies.

### 4.2. Factors Associated with Walking Independence at Hospital Discharge

Logistic regression analysis was used to examine temporal factors associated with walking independence at discharge. The results indicated that none of the clinical assessments conducted 1 month post-stroke onset were associated with walking independence at discharge. At 1 month post-stroke onset, neurological and functional improvement had not yet occurred, and balance recovery was unlikely. Balance ability in patients with stroke involves reduced motor control of the limbs, pelvis, and trunk; sensory impairment; and body spatial cognition. Neurological and functional recovery post-stroke and balance significantly improve between 1 and 3 months post-stroke onset [15]. Therefore, we believe that the Mini-BESTest score may not have been a significant variable in the first month, when recovery of the paralyzed side’s lower limb motor and trunk functions, which are necessary for balance, was in the initial phase.

The Mini-BESTest scores at 2 and 3 months were associated with walking independence at discharge. Specifically, the Mini-BESTest is an assessment index with high accuracy in predicting falls in patients with stroke [34,35]. The BBS, which, similar to the Mini-BESTest, is a measure of balance ability, includes fewer challenging tasks, such as standing without support and maintaining a standing position without support. In contrast, the Mini-BESTest incorporates more complex tasks, such as postural responses to the external environment and balance tasks during walking [35], enabling a more nuanced discrimination of the patient’s walking ability, which was associated with the level of walking independence of patients with stroke at discharge in this study. It also exhibits a floor effect in patients with acute stroke due to the complexity of the task [36]. A previous study examining the Mini-BESTest in patients 60 days post-stroke onset revealed that it showed no ceiling effect in patients with stroke during the recovery phase and was effective in assessing balance ability in participants with good walking ability [22]. In summary, these results underscore the importance of evaluating the Mini-BESTest score over time, commencing 2 months post-stroke onset, in predicting the level of ambulatory independence of patients with stroke at discharge. In the present study, the Mini-BESTest score differed significantly between the groups according to the post-test two-way ANOVA. The Mini-BESTest scores at 2 and 3 months were associated with walking independence at discharge. Previous studies have shown that between 1 and 3 months post-stroke onset is a period of increased neuroplasticity, during which both spontaneous neurological and functional recoveries from rehabilitation interventions are maximized [37]. The results of this study align with those of previous studies and reinforce the importance of rehabilitation from the early stages of stroke onset. Assessing the Mini-BESTest score after the second month of stroke onset provides a detailed picture of the factors contributing to impaired balance and the degree of balance recovery. Balance exercises for patients with poor balance ability after the second month of stroke onset may improve gait independence at discharge. A previous study investigating functional factors influencing the recovery of walking ability in patients with stroke reported that standing postural control was more important than the recovery of support function and voluntary control of the paralyzed lower limb [38]. Therefore, the results of the present study are similar to those of previous studies and suggest that balance ability influences the degree of walking independence in patients with stroke.

### 4.3. Limitations

This study has some limitations. First, physical, balance, and cognitive functions at 3 months post-stroke onset were not investigated. Consequently, we could not confirm an association between changes in physical function, balance capacity, and cognitive function 3 months post-stroke onset and the level of walking independence at discharge. Future studies may yield more rehabilitation-applicable results by predicting gait independence based on long-term changes in physical function up to hospital discharge. Second, this study examined only factors affecting walking independence at discharge in terms of physical and cognitive functions. Future research should explore the influence of brain structure and function in addition to these factors to enable a more accurate prediction of walking independence. Third, this study examined the factors associated with walking independence in patients with stroke at discharge from the hospital, focusing on physical and cognitive functions. Therefore, other confounding factors were not included as adjustment variables. Future studies could include other variables, such as comorbidities and duration of rehabilitation, to provide more accurate predictions. Fourth, the FIM-C has demonstrated interrater reliability and validity in patients with stroke [24]. However, future studies should evaluate cognitive function using other assessment methods, such as the MMSE, which has clearer scoring criteria. Fifth, the sample size was small. Because this study was conducted as a pilot study, its main objectives were data collection and method validation. The sample size was set within the feasibility of a pilot study and was determined based on the practical constraints of data collection and existing resources. It should be noted that future studies will address this limitation by ensuring a sufficient sample size and calculating the statistical power for more accurate analysis. Therefore, caution is required when interpreting the results. Furthermore, the number of participants in future studies should be increased for more precise analysis.

## 5. Conclusions

This study examined the changes in factors associated with walking independence in patients with stroke at hospital discharge. The results revealed that the SIAS-LE, TCT, Mini-BESTest, and FIM-C scores improved significantly over time. The TCT and Mini-BESTest scores were higher in the independent group than in the non-independent group. Additionally, the Mini-BESTest scores at 2 and 3 months post-stroke onset were associated with walking independence at discharge. These results suggest the importance of assessing the Mini-BESTest score over time, commencing 2 months post-stroke onset, in predicting the level of ambulatory independence in patients with stroke at discharge. We believe these findings could contribute to establishing a prediction model of walking independence based on the time since stroke onset, which has not been addressed in previous studies. These results also suggest the importance of using the TCT and Mini-BESTest to assess trunk function and balance capacity when rehabilitating patients between 1 and 3 months post-stroke onset. Early in stroke onset, training to improve trunk function and balance capacity in patients with low TCT and Mini-BESTest scores may improve walking independence at discharge.

## Figures and Tables

**Table 1:** Characteristics of participants.

	All (*n* = 27)		Independent (*n* = 18)		Non-Independent (*n* = 9)		*p*-Value
	Mean	SD	Mean	SD	Mean	SD	
Age (years)	71.74	8.37	71.17	8.55	72.89	8.39	0.62
Height (cm)	156.87	9.27	158.08	9.75	154.44	8.23	0.35
Body weight (kg)	52.96	8.66	53.38	8.65	52.11	9.15	0.73
Length of hospital stay (days)	128.56	5.35	121.11	23.81	138.88	29.18	0.12
Sex: male/female (*n*)	12/15		9/9		3/6		0.68
Stroke type: ischemic/hemorrhagic (*n*)	19/8		14/4		5/4		0.38
FAC at hospital discharge (*n*)							<0.01
0 1 2 3 4 5	0 1 3 5 10 8		0 0 0 0 10 8		0 1 3 5 0 0		

Using the FAC at discharge, the participants were categorized into two groups: 18 in the independent group (9 males and 9 females) and 9 in the non-independent group (3 males and 6 females). The Mann-Whitney U test results revealed that age, height, weight, length of hospital stay, or time of discharge did not differ significantly between the independent and non-independent groups. Additionally, the chi-square test revealed no significant differences in sex, and stroke type at discharge between the two groups; however, a significant difference was observed in the FAC. FAC: functional ambulation category; SD: standard deviation.

**Table 2:** Results of two-factor analysis of variance.

	1 Month		2 Months		3 Months		Interaction Effect Time × Group		Main Effect Time		Main Effect Group		Post Hoc Test	
	Mean	SD	Mean	SD	Mean	SD	F Value	*p*-Value	F Value	*p*-Value	F Value	*p*-Value	Time	Group
SIAS-LE														
Independent	8.33	3.48	10.00	3.38	10.78	3.14	1.62	0.21	13.18	<0.01	2.02	0.17	1 < 2 < 3 m	
Non-independent	7.11	3.62	7.78	4.15	8.33	3.97								
TCT														
Independent	81.94	23.17	92.72	12.93	98.39	4.72	1.42	0.25	22.17	<0.01	7.23	0.01	1 < 2 < 3 m	Non-independent < Independent
Non-independent	56.00	32.00	75.67	23.28	82.89	26.24								
Mini-BESTest														
Independent	6.00	7.10	13.11	7.71	17.83	4.05	1.43	0.25	23.22	<0.01	20.03	<0.01	1 < 2 < 3 m	Non-independent < Independent
Non-independent	0.11	0.33	3.00	5.87	7.78	7.90								
FIM-C														
Independent	28.50	5.19	30.94	3.04	32.33	2.26	0.39	0.69	17.90	<0.01	2.91	0.10	1 < 2 < 3 m	
Non-independent	26.44	4.67	27.89	4.59	30.00	3.57								

No interaction effects between timing and group factors were observed. The SIAS-LE, TCT, Mini-BESTest, and FIM-C scores differed significantly over time. TCT and Mini-BESTest scores differed significantly between groups. SIAS-LE: stroke impairment assessment set-lower extremity; TCT: trunk control test; Mini-BESTest: mini-balance evaluation systems test; FIM-C: functional independence measure cognitive; SD: standard deviation.

**Table 3:** Results of the logistic regression analysis at 1 month.

	B	SE	Wald	df	*p*-Value	Exp(B)	95% CI	
							Lower	Upper
Mini-BESTest	0.58	0.54	1.13	1.00	0.29	1.78	0.62	5.13

Factors associated with walking independence at discharge were not extracted. Model χ^2^ = 8.49, *p* < 0.01. R^2^ (Cox-Snell) = 0.27; R^2^ (Nagalkerke) = 0.38. Hosmer-Lemeshow test χ^2^ = 0.73 (df = 2; *p* = 0.69). SE: standard error; CI: confidence interval; Mini-BESTest: Mini-Balance Evaluation System Test.

**Table 4:** Results of the logistic regression analysis at 2 months.

	B	SE	Wald	df	*p*-Value	Exp(B)	95% CI	
							Lower	Upper
Mini-BESTest	0.19	0.08	6.58	1.00	0.01	1.21	1.05	1.41

The Mini-BESTest score at 2 months was associated with walking independence at discharge. Model χ^2^ = 10.07, *p* < 0.01. R^2^ (Cox-Snell) = 0.31; R^2^ (Nagalkerke) = 0.43. Hosmer-Lemeshow test χ^2^ = 2.85 (df = 4; *p* = 0.58). SE: standard error; CI: confidence interval; Mini-BESTest: Mini-Balance Evaluation System Test.

**Table 5:** Results of the logistic regression analysis at 3 months.

	B	SE	Wald	df	*p*-Value	Exp(B)	95% CI	
							Lower	Upper
Mini-BESTest	0.29	0.11	6.22	1.00	0.01	1.33	1.06	1.66

The Mini-BESTest score at 3 months was associated with walking independence at discharge. Model χ^2^ = 13.34, *p* < 0.01. R^2^ (Cox-Snell) = 0.39; R^2^ (Nagalkerke) = 0.54. Hosmer-Lemeshow test χ^2^ = 3.38 (df = 6; *p* = 0.76). SE: standard error; CI: confidence interval; Mini-BESTest: Mini-Balance Evaluation System Test.

## Data Availability

The data presented in this study are available upon request from the corresponding author. The data are not publicly available due to privacy or ethical restrictions.
